# Malaria Complicating the Puerperium1Read at the semi-annual meeting of the Medical Society of the County of Erie, June 13, 1899.

**Published:** 1899-10

**Authors:** George A. Himmelsbach

**Affiliations:** Buffalo, N. Y.; Assistant Attending Physician, Buffalo General Hospital; Attending Physician, Erie County Hospital


### BUFFALO MEDICAL JOURNAL.

VOL. XXXIX.—LV. OCTOBER, 1899. NO. 3.

### Original Communications.

### MALARIA COMPLICATING THE PUERPERIUM.[1]

BY GEORGE A. HIMMELSBACH, M. D., Buffalo, N. Y.

Assistant Attending Physician, Buffalo General Hospital; Attending Physician, Erie County Hospital.

MALARIA complicating the puerperium is not a new subject, yet from what I have been able to learn from a liberal review of literature, it is one that has not been worn threadbare.

The occurrence of a sudden rise of temperature to 103° or 104° any time during the ten days subsequent to the confinement, is one which sends a thrill of intense anxiety through the mind of the medical attendant, to say nothing of the uncomfortable feeling experienced by the patient, and her newly born as well.

In the event of a sudden rise of fever during the first few days, one's first thought is endometrial infection. We perhaps chide ourselves for erring in our asepsis or antisepsis, and immediately begin a review of our case to determine whether we have committed some gross error in cleanliness, whether the secundines were entirely removed, or whether there has been a relaxation of the uterus with blood clots, intrauterine or intravaginal, that are breaking down. We give our nurse a rigid interrogation as to her method of cleanliness, douching, bathing, activity of the patient's bowels, and the like. All these may be negative, and yet how difficult it is to divert our thoughts away from endometrial infection. So difficult, indeed, that a mistaken diagnosis is too hastily made, and our patient is too often subjected to the unpleasant ordeal of intrauterine cleansing, disturbing a normal endometrium in the process of resolution, and again opening up an avenue of infection.

---

1. Read at the semi-annual meeting of the Medical Society of the County of Erie, June 13, 1899.

I maintain in my paper that, with our modern improved methods of asepsis and antepartum cleanliness, which every physician nowadays carries out, or should carry out, we ought not to ignore the question of parturient infection entirely, but our attention to this particular should be correspondingly lessened, and more thought given to other intercurrent diseases, which may be mentioned as causes of pyrexia during the puerperal period, such as pleurisy, pneumonia, rheumatism, any of the acute exanthemata, intestinal toxemia, breast infection, endo- or pericarditis and malaria.

I think we can consider malaria as one of the most important intercurrent diseases of the puerperium, not only because women recently confined have an increased susceptibility to the disease, a fact now generally conceded, but especially because the disease, so often simulates sepsis, from which it is of the utmost importance that it be differentiated.

We are told that women who have been residents of a malarial climate, and who have the plasmodium lurking in their systems, and yet have no manifestations of the disease, almost always suffer from paroxysms following labor; whether this is the direct result of the traumatism of the labor or hemolytic changes, the result of blood loss, is still a mooted question.

The title of this paper was suggested to my mind from the fact that I have met with several undoubted cases of malaria, complicating the child-bed, in my own practice. Judging from this, I thought that an abundance of material could be found from which to study, and to incorporate in this paper, but it has been a surprise to me, after a thorough search of current literature of the past ten years, and even our latest text-books, to find the scarcity of references to this subject. Upon interviewing men of wide obstetric experience, in our own city, I am told of the frequency of its occurrence, and the too often error in diagnosis, mistaking it for infection of the parturient canal. One writer says he has knowledge of a case in which a high fever developed after childbirth, and the obstetrician in charge supposing the fever to be septic, curetted and irrigated the uterus, without benefiting the patient's condition. It was discovered later, the woman had a croupus pneumonia. Similarly, I say a woman may be curetted unnecessarily who suffers from malaria, rheumatism or any other febrile disease.

J. D. Rowlings, *Lancet*, August 7, 1897, says that if in the first three days of the puerperium, the temperature rises without obvious cause to 102°F. and is accompanied by a pulse rate of 120 or more, the case should be regarded, provisionally, as septic. This may be good advice, and in a measure true, yet, note how guarded he is in saying "without obvious cause" and "provisionally septic." I say even at or before the third day we should not forget the possibility of malarial infection as a cause of pyrexia.

M. Salowieff, in a French journal of June, 1894, relates the case of a woman suddenly attacked by an intense fever at the end of her pregnancy; of another in whom the fever came on two weeks after the delivery, and of a third who was affected about the middle of her pregnancy. In all cases the treatment with quinine and opium associated with arsenic was effective. He further states that malaria may be the cause of abortion, premature delivery, or in some cases death of the fetus. In those recently delivered it interferes with the milk secretion. He attaches great importance to the diagnosis of this fever from other affections of the child-bed, especially from puerperal septicemia.

The diagnosis and differential diagnosis, is sometimes very difficult, and it is a safe rule to reserve your opinion until you have made thorough enquiries into the case and have satisfied yourself that the parturient canal is unaffected.

The appearance of the temperature curve is very suggestive; as a rule, the first chill and pyrexia appear on the third day following the confinement; this, however, is not always so; they may come at any time during the period.

The duration and marked remission of the fever, and the quite regular periodicity, with a pulse the frequency of which keeps pace with the temperature curve are diagnostic. It is sometimes quite difficult under the circumstances to examine the spleen, but we are told that it is invariably enlarged.

The subjective examination of our patient is especially important. We should arm ourselves with the history of her previous health; whether she has ever had an attack of malaria, even though remote, perhaps years previous, for I was told by a prominent obstetrician sometime ago, that he was of the firm conviction that a person once a sufferer from this disease, never, in spite of heroic antimalarial treatment, and in spite of no apparent manifestation of the disease, the organism never becomes thoroughly eradicated, and that it exists throughout life. This I think is quite generally accepted.

A thorough investigation as to the sanitary condition of the surroundings should be made as to whether there are pools of stagnant water in the cellar, or under the house, where no cellar exists ; or in vacant property adjoining; whether there has been any recent excavation of soil for cellar building, for street paving, for laying new sewers, gas or water mains. These are of importance.

Finally, two great diagnostic points in malaria, suggested by Osler, in a paper on this subject in 1896, are the invariable association of the plasmodium of Lavaran and the invariable curative effects of quinine.

In the *Archives de Toxocologie*, January, 1892, Aberlin reports a study of forty-six cases of malaria complicating the puerperium. He believes that in order to be certain that febrile attacks in the puerperium are due to malaria, it is necessary to exclude all causes of high temperature due to local disturbances of the genitals and the breasts. He believes that malaria is not more severe during the puerperium than at any other time, and if treated from the start is not a serious complication.

Sewell, in the Cincinnati *Lancet Clinic*, of January, 1892, relates a case of intermittent malarial fever, which developed twenty-four hours post partum.

Miller, in the Kansas City *Medical Index*, May, 1892, reports a case of chronic malarial poison, complicating the puerperal state, in which tetanus developed.

I herewith append memoranda of several of my own cases :

Case I.—Mrs. G., age 30 years, primipara, confined July 15, 1895, labor normal; secundines removed entire; contractions good; puerperium uneventful until July 30th (14 days) when taken with severe chill, followed by high fever, and general feeling of malaise and discomfort. Examination discovered two sloughing ulcers, one on each labium at vaginal entrance. Some necrosis of soft parts. She complained of vesical tenesmus and a urinalysis discovered pus cells present—but these might have come from urine passing over the external wounds.

Counsel was called and we decided to curette and irrigate. This was done, but nothing was found intrauterine, sufficient to account for the pyrexia. The following day she again had a chill about the same time, followed by high temperature, and again the third day. Dr. Frederick saw the case with me and suggested the administration of quinine in fifteen or twenty grain doses. This therapy was continued for ten days or so, and completely controlled the paroxysms, except on the fifth or sixth day, when the drug was intentionally omitted, which resulted in the recurrence of another paroxysm of rigor and temperature, but much milder than the previous attacks.

The treatment in this case proved conclusively the diagnosis of malaria. A thorough search of the premises was made, there being no antecedent history of the disease, and it was found that in the vacant property adjoining the house in which my patient resided, were pools of stagnant water, which had stood for a long time prior to her confinement.

Case II.—Mrs. H., age 31 years, multipara, delivered November 27, 1896, third confinement, which was six hours long; normal; secundines removed intact; uterine contractions good; puerperium normal until December 1st, when taken with a chill, followed by fever; this occurred three days in succession and each succeeding day more intense than the previous one; the third was a very severe rigor. Lochia was normal in amount and odor. By a process of exclusion I satisfied myself I had to deal with a case of malarial infection. Quinine sulphate was given in large doses, with no return of the paroxysms. I learned upon examination that the street in which my patient resided had been recently excavated and torn up from end to end, for the purpose of laying large (two foot) gas mains and for paving the street. This history coupled with the fact of controlling the paroxysms with quinine sulphate, confirmed my diagnosis.

Case III.—Mrs. M., age 22 years, primipara, confined March 27, 1897. About the seventh month of her pregnancy she consulted me complaining of feeling poorly the latter part of each day. Said she experienced peculiar flashes pass over her body, which made her feel so miserable at the time she was obliged to lie down; she saw me several times during the next few weeks; a bimanual examination was made—negative. The urine normal. At the beginning of her ninth month she called and said she had nearly reached the limit of her endurance. The flashes had developed into distinct chills and each one followed by fever, headache, backache and general depression.

I now mistrusted what I had to deal with. She denied ever having had malaria, that she was aware of, but said that soon after her marriage she moved into a new house just completed, where the plaster and wood-work were still green. To satisfy my curiosity I called at her home, and to my surprise I found the house actually wet (not simply damp.) In some rooms, which were prettily frescoed and papered, the latter was falling from the walls in sheets. The cellar was in a most unwholesome wet state, water standing about in pools and the ground was soft and "knee deep."

My patient was immediately given quinine sulphate in large doses, against the alleged action of this drug as an ecbolic. In a few days she felt entirely well. I gradually withdrew the drug as the date of her confinement drew near. All went well until the fourth or fifth day after her confinement, when the plasmodium again began to assert itself in the form of slight chills and elevation of temperature. She was again put upon quinine and continued for sometime with no further manifestations of the disease.

Case IV.—Age 24 years, seven to eight months pregnant; taken with chilly sensations, followed by high fever on Wednesday, May 17, 1899. This occurred again on the next day and the third day (Friday) with more severity, and she now began to feel in addition with the chills and fever, vague pains in her bones and body generally. On Saturday I saw her with a high fever, following a chill and pains resembling labor pains. On Sunday I was summoned and found her with a temperature of 103°; pulse, 110; recurring pains and os dilating. On the evening of same day I delivered her of a premature child—probably seven to eight months. The following day she had a chill with temperature 103° and again the second day following confinement; lochia normal. I administered quinine sulphate, in ten grain doses every day, and in a few days chills and fever all disappeared. She informed me that a year previous, while living in Detroit, she suffered two months with malaria and was on quinine treatment for a long time. I believe in this case the diagnosis of malaria ante- and post partum was correct, based on the previous history of malaria and control of paroxysms with quinine; I believe, too, that the malaria was in a large measure responsible for the premature confinement.

In conclusion let me say that a favorable prognosis of a given case, depends upon a correct interpretation of symptoms and an early recognition of the disease.

Therapeutics properly directed will mitigate symptoms at once, and save an otherwise protracted siege of chills and fever and their usual sequelæ.

137 W. Tupper Street.

